# Comorbidity Matters: Social Visual Attention in a Comparative Study of Autism Spectrum Disorder, Attention-Deficit/Hyperactivity Disorder and Their Comorbidity

**DOI:** 10.3389/fpsyt.2020.545567

**Published:** 2020-09-30

**Authors:** Chara Ioannou, Divya Seernani, Maria Elena Stefanou, Andreas Riedel, Ludger Tebartz van Elst, Nikolaos Smyrnis, Christian Fleischhaker, Monica Biscaldi-Schaefer, Giuseppe Boccignone, Christoph Klein

**Affiliations:** ^1^Department of Child and Adolescent Psychiatry, Psychotherapy and Psychosomatics, Medical Faculty, University of Freiburg, Freiburg, Germany; ^2^School of Psychology and Clinical Language Sciences, University of Reading, Reading, United Kingdom; ^3^Department of Psychiatry and Psychotherapy, Medical Faculty, University of Freiburg, Freiburg, Germany; ^4^Department of Psychiatry, National and Kapodistrian University of Athens, Athens, Greece; ^5^Department of Computer Science, University of Milan, Milan, Italy; ^6^Department of Child and Adolescent Psychiatry, University Hospital Cologne, Cologne, Germany

**Keywords:** autism spectrum disorder, attention-deficit/hyperactivity disorder, comorbidity, neurodevelopmental disorders, eye tracking, social cognition

## Abstract

Autism Spectrum Disorder (ASD) and Attention-Deficit/Hyperactivity Disorder (ADHD) represent two common neurodevelopmental disorders with considerable co-occurrence. Their comorbidity (ASD + ADHD) has been included in the latest diagnostic guidelines (DSM-V, 2013). The present study focuses on social visual attention that i) is a main aspect of social attention reflecting social cognition and ii) its atypicalities have been suggested as a potential biomarker for ASD. Considering the possible shared background of both disorders and their comorbidity, it is important to compare such traits directly. Here, 73 children and adolescents paired for age and IQ diagnosed with ASD (N = 12), ADHD (N = 21), comorbid ASD + ADHD (N = 15), and “typically developing” (TD) controls (N = 25), were shown static real-life social scenes while their gaze movements were recorded with eye-tracking. Scenes with two levels of social complexity were presented: low complexity (one person depicted) and high (four interacting individuals). Gaze fixation variables were investigated. Fixation duration on faces was significantly reduced only in ASD + ADHD which also required longer time to fixate all faces at least once. Fixation duration on faces in ASD was reduced, compared to TD, only when looking at scenes with high versus low social complexity. ADHD individuals did not differ from TD. Concluding, the observed alterations of social visual attention support the existence of possible dysfunctional particularities differentiating ASD, ADHD, and ASD + ADHD, which can be revealed with the new method of eye-tracking technique. The objective gaze measurements provided contribute to the development of biomarkers enabling early diagnosis, amelioration of care and further interventions specified for each group.

## Introduction

Autism Spectrum Disorder (ASD) and Attention-Deficit/Hyperactivity Disorder (ADHD) are two of the most studied Neurodevelopmental Disorders (1). The main diagnostic criteria of autism include qualitative impairments in social communication and interaction, restricted repetitive patterns of behavior and hyper- or hyporeactivity to sensory aspects of the environment, while those of ADHD consist mainly of inattention, impulsivity and hyperactivity (2). The prevalence of both disorders has risen both in industrial and in low-income countries to a globally estimated 1% or higher for ASD and 2%–7% for ADHD (3–5). Interestingly, ASD and ADHD are frequently co-occurring with an estimated 30%–80% of ADHD in the autistic population and 20%–50% of ASD in the ADHD population (6). Importantly, the latest diagnostic criteria of DSM-V have allowed their simultaneous diagnosis (2).

This co-occurrence has been under discussion both for clinicians and researchers even before DSM-V implemented the changes, in relation to symptom manifestation, both of autistic traits in ADHD individuals (7) and vice versa (8), as well as comparisons of the two disorders concerning their social deficits (9, 10), their neurophysiological similarities and the potential aetiological and biological overlap (11, 12). However, for the most part, research studies of the ASD samples have not controlled for comorbidity with ADHD. This ambiguity in sample definitions might have resulted in misinterpretations of ASD or ADHD traits and in reduced awareness of the specific characteristics of the comorbid population (from now on referred to as ASD + ADHD). Particularly, social cognition traits that highly affect the quality of life of both ASD and ADHD (13, 14) are thought to be more severely impaired in comorbid ASD + ADHD cases (15). Also, comorbid individuals seem to respond differently to existing interventions as they cause challenges in medical treatment (16) and may benefit less from social skills trainings (17). Finally, comorbid cases have been proposed to present a more complex phenotype with more severe deficits in the clinical and cognitive domains, including social deficits (18). Taken together, there is a need for valid biomarkers characterizing and differentiating ASD, ADHD, and ASD + ADHD groups (19).

Social cognition traits represent a main domain of shared and unique characteristics in ASD and ADHD (12). The two disorders share similar social cognitive deficits (9, 10), but their comorbidity may also reflect representative social features of each disorder (20). Interestingly, the link between social ineptness and inattention has been suggested as a pathway which could explain the co-occurrence of ASD and ADHD (21). Under this perspective, the functions combining these elements, such as attention to social information, are worth investigating. Concretely, as defined in a recent conceptual review, visual social attention composes together with social motivation and social behavior, the three core aspects of social attention (22) and can be examined with the eye tracking technique (23). Due to its non-invasiveness and suitability for testing young and/or intellectually impaired individuals, this technique has been applied in neurophysiologic and psychiatric research (24–26). The Free Viewing Task is one of the basic eye-tracking paradigms in social cognition, where the participant is viewing social scenes without specific instructions while his/her gaze is recorded.

In ASD research, various eye-tracking studies including participants from infancy until adulthood exist (27–30). Particularly, in children or adolescents, some studies report that ASD participants look less to the faces of social scenes compared to Typically Developing (TD) peers (31, 32), whereas other studies report similar performance of TD and ASD (33, 34). In some such cases, the ASD participants, however, needed longer to fixate on faces (35). These inconsistencies are addressed by seminal review articles, concluding that social attention in ASD participants is most impacted when stimuli have a high social content, namely, when stimuli show more than one person (33, 34) and human interactions (36). Importantly, this criterium is proposed as a discriminating factor differentiating ASD from TD and is referred to as social complexity (37). The level of social complexity of the depicted scenes is thus defined as the number of people in the scene and the degree of social interaction between them. Accordingly, higher social complexity of a scene should reveal atypical visual attention in ASD participants in terms of reduced attention at social elements.

Compared to the ASD literature, a limited number of studies have emerged regarding ADHD and social visual attention (38). The few existing studies focus mainly on isolated face pictures and report deficits in facial emotion recognition (39) or reduced looking preferences for the eye region (40). Moreover, in non-eye tracking studies, ADHD shows deficits in theory of mind, prosody perception, and empathy (41) and it is reported that deficits in social cognition may impact their symptomatology altogether (42). However, others suggest that children with ADHD are not as impaired as children with ASD on theory of mind tasks (43). Thus, there is not enough evidence that social attention deficits occur when looking at real-life social scenes.

Even less is known about participants with comorbid ASD and ADHD and to the best of our knowledge, there have been no eye-tracking studies about the visual exploration of social scenes in this group. However, it has been reported that ASD + ADHD participants showed deficits in attentional orienting as measured with Event-Related Potentials of face and gaze processing (44). Moreover, a behavioral social cognition study without eye-tracking suggested that children with comorbid ASD + ADHD are at highest risk for emotion recognition problems in comparison with TD, ASD, and ASD-unaffected siblings (45). Interestingly, the scope of this approach was to investigate whether emotion recognition may represent an endophenotypic candidate for ASD while evaluating the impact of comorbid ADHD (45).

Eye tracking measurements represent accurate, reproduceable measures that might serve as potential objective indications of medical states in order to predict the incidence and early diagnosis of a disorder as well as monitor outcomes of interventions (46). Regarding ASD, visual attention to social relevant stimuli has been recently suggested as a promising early behavioral biomarker (47). In addition, considering the high comorbidity of the ASD and ADHD and their suggested aetiological and biological overlap (48), comparisons between all respective clinical groups are necessary for the development of such valid biomarkers.

The present study sets out to fill the described gap by directly comparing the ASD, ADHD, and comorbid ASD + ADHD groups in social visual attention to real-life scenes with different levels of social complexity. The study design enables us to differentiate the following scenarios: whether altered social visual attention measures are (a) specific to ASD and thus absent in those with ADHD symptoms; (b) present in ASD and more pronounced in comorbid cases; (c) a shared feature of these neurodevelopmental disorders. Additionally, interactions with the level of social complexity, early gazing behavior, and latencies until first facial fixations are explored.

## Material and Methods

### Participants

Children and adolescents were recruited from four predefined groups: TD, ADHD, ASD, and ASD + ADHD. Inclusion criteria were normal or corrected-to-normal vision, intelligence quotient (IQ) ≥70, native-speaker level in the local language and age between 10 and 14 years. The minimum required age was chosen, since participants had to read for one of the overall study’s tasks, while the age span was kept relatively short in order to reduce the developmental bias. Subjects with strabismus, clinical diagnoses of Tourette syndrome, specific reading disorder, epilepsy, schizophrenia, depression or any known specific genetic syndrome associated with ASD or ADHD were explicitly excluded. Finally, 73 out of the 89 recruited children and adolescents aged 10–14 years were included (TD: N = 26; ADHD: N = 26; ASD: N = 16; and ASD + ADHD: N = 21; see *Results*), paired for age and IQ [for power calculations see (49)].

TD participants were recruited from local schools, sports groups and the institutional database including children or adolescents that had participated in previous unrelated neuropsychological studies and had expressed interest in participating in further studies. Participants for the clinical groups were recruited from the department database. The diagnoses were made prior to the study by three experienced child- and adolescent psychiatrists (with 22, 16, and 14 years of experience) and three clinical psychologists (with 19, 12, and 10 years of experience) based on the ICD-10 criteria (in our study F84.0, childhood autism; F84.1, atypical autism; F84.5, Asperger’s syndrome; F90.0, predominantly inattentive type ADHD; F90.1, predominantly hyperactive type ADHD). Diagnoses were verified before recruitment.

ASD was diagnosed based on the gold standard for autism diagnosis, namely, the self-rated Autism Diagnostic Observation Schedule [ADOS (50)] and the parent-rated semi-structured Autism Diagnostic Interview [ADI-R (51)]. These measures, especially in combination, are suggested to have the largest sensitivity and specificity (52). Status of previously accomplished social competence training, consisting of 12 hourly modules, was extracted from the database of our department for the ASD and ASD + ADHD groups (53). Particularly, the social competence training used, the so-called “Theory-of-Mind-Training in Autism Spectrum” [TOMTASS (54)], is an established method based on behavioral therapy and specified autistic intervention, namely, the program “Treatment and Education of Autistic and Related Communication Handicaped Children” [TEACCH, (55)]. In our study, it was applied in local language, in a time period of five months, in small groups of three participants. The diagnosis of ADHD was based on: i) the Conner’s parent and teacher rating scales (56); ii) comprehensive interviews and history with the child’s adult caregivers; iii) comprehensive interviews and behavioral observation of the child. This diagnostical procedure is in alignment with recommendations for reliability of diagnosis (57).

For the purpose of this study, assessments of general and specific symptomatology in native language were applied additionally, characterizing the semiology of each group. Assessment of general emotional and behavioral problems was realized for all participants with the parent-rated Child Behavior Checklist (CBCL/6-18) (58), which has been proved valid in screening of ADHD (59). Specific assessment of autistic semiology was performed across all participants using the school-age Social Responsiveness Scale (SRS), a quantitative measure of autistic traits applicable in both clinical settings and large-scale research studies of ASD conditions (60). For assessment of ADHD semiology, the established parent-rated external assessment questionnaire (EAQ) and the self-assessment questionnaire (SAQ) questionnaires based on the ICD-10 and the DSM-IV were used in native language (61). All participants were asked to be medication-free from psycho-stimulants for at least 24 h before testing (62). Medication intake of the clinical groups is listed in **Table 1**.

**Table 1:** Demographics, assessment and diagnostic scoring of the distinct groups.

Variable	TD		ADHD	ASD	ASD + ADHD	Statistical inference	
						Hypothesis testing	contrasts
Number of participants	25		21	12	15	–	–
Males (in %)	52		76	83	93	χ^2^ = 9.4 p = 0.02	–
Age in years	12.1 ± 1.5 (10.6–13.6)		12.6 ± 0.9 (11.9–13.1)	12.3 ± 1.1 (11.7–13.0)	12 ± 1.0 (11.4-12.8)	p = 0.48	–
IQ	110 ± 17 (96–122)		102 ± 13 (92–111)	103 ± 21 (88–114)	96 ± 15 (84–109)	p = 0.07	–
Education level 1, 2a, 2b, 2c (in %)^#^	8, 0, 40, 52		0, 10, 76, 14	8, 25,42,25	20, 7, 7, 66		
Social training (in %)	–		–	50	40	χ^2^ = 0.02 p = 0.89	
CBCL-total problems	50 ± 6.6 (46–55)		64.9 ± 7.9 (59–72)	68.1 ± 8.3 (64–74)	64.7 ± 8.5 (62–70)	p <.001	TD < ADHD, ASD, ASD + ADHD
SRS-total	44.2 ± 8.7 (40–48)		66.6 ± 10 (62.5–71)	76.6± 13.4 (67–83)	77.0 ± 8.4 (72–78)	p <.001	TD < ADHD < ASD, ASD + ADHD
ADHD symptomatology							
EAQ-total	–		7.1 ± 1.1 (6.5–7)	6.6 ± 1.1 (6.8–7)	7.2 ± 0.9 (6.9–7.1)	p = 0.28	–
EAQ-Competence*	–		3.4 ± 1.2 (3–4)	4.8 ± 1.6 (4–5.5)	4.1 ± 1.4 (3.3–5)	p = 0.02	ADHD < ASD
SAQ-total	–		6.3 ± 1.8 (5–8)	5.5 ± 1.6 (4–7)	6.8 ± 1.3 (6–8)	p = 0.13	–
SAQ-Competence*	–		4.9 ± 1.8 (4.3–6)	5.8 ± 1.1 (5.8–6.3)	5.3 ± 1.7 (5–6)	p = 0.29	–
ADOS total score	–		–	12.6 ± 4.9 (10–17)	10.2 ± 4.3 (7–13)		
ADI-R interaction	–		–	12.6 ± 4.9 (10–17)	12.6 ± 4.9 (10–17)		
ADI-R communication	–		–	13.8 ± 5.1 (11–14)	15.6 ± 7.6 (10–22)		
ADI-R restricted and repetitive behavior	–		–	5.2 ± 2.9 (8–13)	4.8 ± 3.1 (3–6)		
Permanent medication (count)	–		MPH 12^†^	AAP 1	AAP 2, MPH 6^†^		

Values correspond to group mean ± 1 standard deviation, while 25^th^ percentile and 75^th^ percentile are inside parentheses, if not stated otherwise. All scores for CBCL and SRS are T-scores, while all scores for EAQ and SAQ are Stanine scores. ^#^Education level is categorical (1: primary school, 2a–c: secondary level, lowest to highest sublevels). *Higher competence scores are associated with normal functioning. ^†^Medication had been discontinued at the day of the experiment. Abbreviations: TD, Typically Developing; ADHD, Attention-Deficit/Hyperactivity Disorder; ASD, Autism Spectrum Disorder; ASD + ADHD, comorbid group with ASD and ADHD; χ^2^, chi-squared; p, p-value; “<“ and “>“ symbols indicate statistically significant contrasts; CBCL, Child Behavior Checklist; SRS, Social Responsiveness Scale; EAQ and SAQ, External- and Self-assessment Questionnaires for ADHD, respectively, based on ICD-10, DSM-V (39); ADOS, Autism Diagnostic Observation Schedule; ADI-R, Autism Diagnostic Interview-Revised; MPH, methylphenydate; AAP, atypical antipsychotics.

The local institutional ethics committee approved the study protocol, which was conducted in accordance with the Declaration of Helsinki (63). All participants and their parents/legal guardians were comprehensively informed about the purpose and the procedures of the study and gave their written consent before participating.

### Procedure

**Figure 1:**
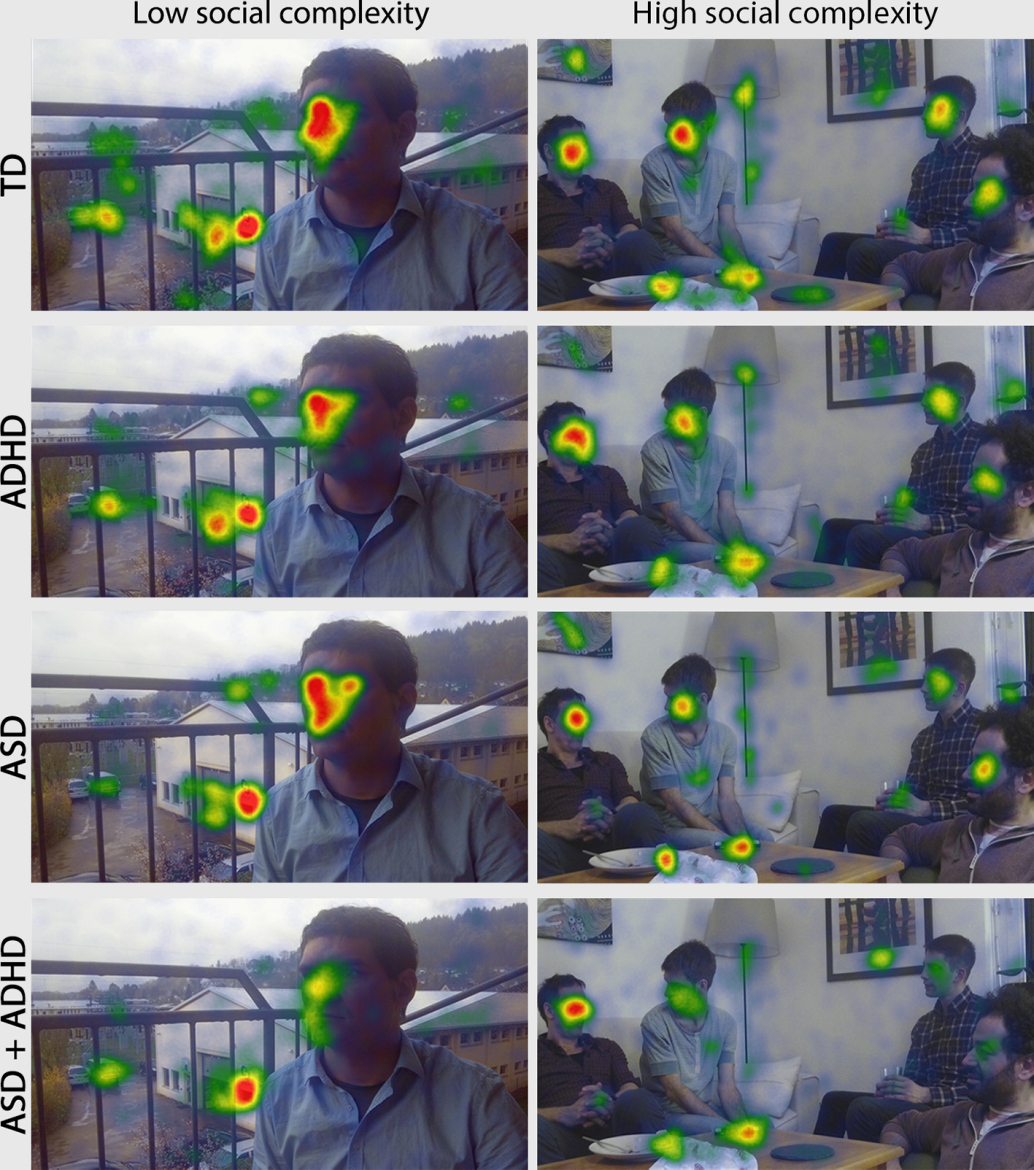
Heatmaps representing the amount of fixation duration for each group and stimuli of high and low social complexity. The warmer the color of the superimposed heatmap, the higher the fixation duration, while the scale is common for all subfigures. The ASD + ADHD group looks on average less at faces in both levels of social complexity, while ASD participants look less at faces when looking at the social complex picture with four interacting people rather than in the picture of low social complexity with one face only. The ADHD participants look at faces similar to TD. Abbreviations: TD, Typically Developing; ADHD, Attention-Deficit/Hyperactivity Disorder; ASD, Autism Spectrum Disorder; ASD + ADHD, Autism Spectrum Disorder with comorbid Attention-Deficit/Hyperactivity Disorder.

The experimental procedure was composed of two sessions, for the majority of the participants in following order: in the first session (60 min, including breaks) an IQ test was performed [Culture Fair Intelligence Test 20, CFT 20-R; (64)] and previously distributed questionnaires were collected. The second session (a battery of six eye-tracking tasks totalling 90 min, including breaks) was conducted in a sound-attenuated Faraday cage, while an experimenter provided instructions and observed the participants from a lateral-rear position. A second experimenter monitored the gaze attention and eye trackability of the participant throughout the whole experiment. Participant-screen distance was 70 cm. Participants were instructed to avoid unnecessary head movements or speaking during the task and a comfortable position was ensured without chin-/forehead rest. The various tasks of the experiment, including the Free Viewing task, were presented in counterbalanced order across participants of each group. After successful calibration, as described below, participants were presented with static stimuli of different social complexities in fixed order with a run duration of 2 min. These consisted of four original pictures of real-life scenes, two with one person and two with four people interacting, representing low versus high social complexity stimuli, respectively (see **Figure 1**). Their content and scenery was developed based on existing literature and the International affective picture System (IAPS) (65) with participation of professional actors. For both levels of social complexity, the scenes take place both indoors and outdoors. All scenes represent everyday situations: i) the social interactions of four individuals show people sitting and discussing, and ii) the single-person scenes show an individual from a frontal or side view. Details regarding selection criteria and scene content are listed in Supplementary Materials S1. In order to minimize the effects of workload and fatigue, participants were mostly tested on weekends or during school holidays and had adequate breaks between tasks, if needed.

Eye tracking stimuli were presented on a 24”-TV Monitor (screen resolution of 1,920 × 1,080 pixels and screen refresh rate of 60 Hz) with the Presentation^®^ software (Version17.2, Neurobehavioral Systems, Inc., Berkley, CA). For data recording the iView RED250 remote eye tracker (SensoMotoric Instruments GmbH, SMI) was used with a sampling frequency of 120 Hz and a visual angle of 20° and binocular tracking mode. Preceding each task, a standard five-point calibration and four-point validation was conducted, as suggested by the software’s manufacturer, which was considered as successful if the deviance was lower than 0.5° (66). Event classification in fixations, saccades or blinks and data export was accomplished with the SMI software *BeGaze 3.7*, excluding off-screen fixations and/or blinks. The default software thresholds for identification of fixations, defined as a gaze event of minimum 60 ms and a maximum dispersion of 2°, were applied (67).

In addition to the above-mentioned selection criteria, data were examined separately in real time speed and only data fulling following thresholds were included: tracking ratio >75% (percentage of non-zero gaze positions divided by sampling frequency multiplied by run duration) and/or a total fixation duration ratio >50% (percentage of total fixation duration divided by run duration), as previously described (68, 69).

### Data Analysis

Areas of Interest (AOIs) were delineated for every picture annotating the whole image and differentiating the following categories: face, body and non-social, the latter including all non-social elements of the stimulus (i.e., objects). This categorization on naturalistic scenes, including images of people during an interaction, has been used in previous studies (70). Eye tracking measurements were exported for each subject and AOI from the SMI Software and heatmaps of fixation duration were generated for quality control. Fixation data were further processed with MATLAB (R2018b, The MathWorks, Natick, MA, USA) for the calculations described below.

#### Fixation Durations

Two basic types of fixation duration variables were calculated (in seconds, s) for each participant: i) total fixation duration representing the sum of fixation durations over one stimulus, irrespective of AOI and ii) fixation durations for each AOI category, defined as the sum of fixation durations for each stimulus and representing the total fixation duration spent respectively in the three AOI categories. For the face AOIs, the analysis was additionally conducted for the first 5 and 10 seconds, representing the early gazing behavior of the visual exploration (33).

#### Latencies to Face Fixations

Additionally, two latency variables were investigated for the face AOIs (in seconds) (70). Firstly, the “latency to first face fixation”, defined as the time required until the first fixation to a face AOI. Secondly, only for the stimuli of high social complexity, the “latency to completion of face fixations” was defined as the time period required until all four interacting faces were fixated for the first time. Former variable has been used before (70), while latter was introduced here in order to capture the time required for visually exploring all faces and not only one of them.

### Statistical Analysis

Statistical analysis was conducted with the statistical software R (R Core Team, v3.6.1). Linear mixed effects models were implemented for the analysis of the interaction between fixation duration and clinical groups with the “lme4” library (v1.1-19) (71), while p-values were obtained by the “lmertest” library with a significance threshold defined as p-value < 0.05. In general, small sample sizes are tolerated with linear mixed effects models, as this type of linear regression accounts both for by-item and by-subject variation in a single model (fixed and random effects, respectively), the latter also being an important advantage when applying to psychiatric populations. Based on the described benefits, linear mixed effects analyses are strongly recommended and their application is rapidly increasing (72), while they have been recently introduced in eye-tracking data analysis (40) and neurophysiological studies (73).

Firstly, total fixation duration was examined and following fixed effects were entered into the model: i) group of participants (TD, ADHD, ASD, ASD + ADHD), ii) level of social complexity (high, low), iii) interaction term between group of participants and level of social complexity, and iv) status of previous social training (yes, no). A random intercept was used for each subject. Secondly, the fixation duration of each of the three AOI-categories (face, body, and non-social elements) was examined with separate linear mixed effects models, structured accordingly. Here, the same explanatory variables as above were introduced as fixed effects, while individual total fixation duration for the respective stimulus was added as a covariate, in order to limit the effect of inter-individual differences in total fixation duration. Finally, similarly to the analysis of the whole run duration, the fixations to the faces were analysed separately for the first 5 and 10 s with total duration of fixations in the respective timespan as a covariate.

For the latency variables two-way analysis of variance (ANOVA) was implemented, with the group of participants as the between-subject factor and stimuli as the within-subject factor. As only one value is available per stimulus, mixed effects models cannot be applied. For the statistical analysis of demographics, an ANOVA was used for continuous variables and a chi-square (χ^2^) with Yates’ continuity correction for categorical variables.

## Results

From the recruited participants three did not complete all sessions (1 ADHD, 2 ASD + ADHD), three did not fulfil the IQ criterion (1 ADHD, 1 ASD, and 1 ASD + ADHD) and one ADHD patient had not discontinued medication, leading to their exclusion from the analysis. Datasets of further nine participants with subthreshold values of tracking ratio and/or total fixation duration ratio (1 TD, 2 ADHD, 3 ASD, 3 ASD + ADHD) were excluded. Finally, datasets from n = 73 participants were included in this present study. Groups did not differ in age and IQ (mean ± standard deviation for age: 12.1 ± 1.5, 12.6 ± 0.9, 12.3 ± 1.1, 12 ± 1.0, p-value 0.48; and for IQ: 110 ± 17, 102 ± 13, 103 ± 21, 96 ± 15, p-value 0.07 for TD, ADHD, ASD, ASD + ADHD, respectively). There was a male predominance in the clinical groups (sex-ratio 52%, 76%, 83%, 93%, respectively). Previously accomplished social training in the ASD groups was equally balanced (50% and 40% for ASD and ASD + ADHD, respectively). In the ADHD group both main types were represented (N = 13 with F90.0 and 8 with F90.1). In the ASD group childhood autism and Asperger’s syndrome were the main diagnoses (N = 3 with ICD-10 F84.0 and N = 8 with F84.5, respectively), while 1 subject was diagnosed with atypical autism (F84.1). In the ASD + ADHD group the distribution was similar (N = 4 with F84.0, N = 9 with F84.5 and N = 2 with F84.1) and regarding comorbidity, either it was diagnosed as such, or the criteria for both ASD and ADHD were fulfilled (for those diagnosed before the introduction of DSM-V). Confirmed diagnoses, as well as ADOS and ADI-R, are listed at the bottom of **Table 1**. Reported comorbidities of included participants are listed in the **Supplementary Table 1**.

Descriptive statistics of diagnostic tests, questionnaire scoring results and results of statistical inference, as well as sociodemographic variables are shown in **Table 1**. TD participants showed T-scores inside the reference range (values below 60 are categorized as normal) in the CBCL-total problems (50 ± 6.6) and the SRS-total score (44.2 ± 8.7). Clinical groups showed average T-scores above the cut-off in the CBCL-total and SRS-total scoring (values above are categorized as borderline or clinical see **Table 1**). Specifically, concerning the scoring of autistic symptomatology, the ASD and ASD + ADHD groups did not differ significantly but showed a statistically significant higher SRS-total score (76.6 ± 13.4 and 77.0 ± 8.4, respectively, values above 75 indicate severe social impairment) compared to the TD and ADHD group (44.2 ± 8.7, p-value < 0.001 and 66.6 ± 10, p-value < 0.05, respectively). Finally, in the scoring of ADHD symptomatology, the ADHD and ASD + ADHD groups did not differ statistically in the Stanine-scores (7.1 ± 1.1 and 7.2 ± 0.9, respectively), which were above average (meaning a Stanine > 7) in the EAQ total score and showed lower values in the competence scores compared to the ASD (3.4 ± 1.2 vs. 4.8 ± 1.6, p-value < 0.02).

### Total Fixation Duration

For the stimuli with low social complexity, the control group showed a total fixation duration of 109 ± 7.1 s (mean ± standard deviation, SD), while the clinical groups showed lower total fixation durations: the ADHD group 104 ± 11.6 s, the ASD group 103 ± 12 s and the ASD + ADHD group 103 ± 10.6 s. For the two stimuli with high social complexity, the control group showed a mean total fixation duration of 104 ± 9.8 s, while the ADHD group spent 103 ± 7.6 s fixating, the ASD 100 ± 12.3 and the ASD + ADHD 98 ± 13.1 s. Boxplots did not show any extreme outliers.

In the respective mixed effects analysis, the total fixation duration was significantly longer by 3.6 s for all groups at the stimuli with low socially complexity (F-value = 15.5, p-value of *post-hoc* test < 0.001). For the ASD + ADHD group and both levels of social complexity, total fixation duration was significantly lower by 6.2 s (F-value = 1.7, p-value of *post-hoc* test = 0.038, see **Supplementary Table 2**). There was no significant interactive effect between social complexity of stimuli and group of participants or effect of previous training of social competence on total fixation duration. Inspection of residual plots did not reveal any obvious deviations from homoscedasticity or normality.

### Fixation Duration on Faces

Across all groups the average of total fixation duration on faces was 21 ± 9.9 s for low social-complexity stimuli and 7.1 ± 4.7 s for high social-complexity stimuli, as in the latter stimuli the fixation duration was distributed among the four depicted face AOIs. **Figure 1** shows fixation distribution representations (heatmaps) for participants of all groups in two levels of social complexity.

**Figure 2:**
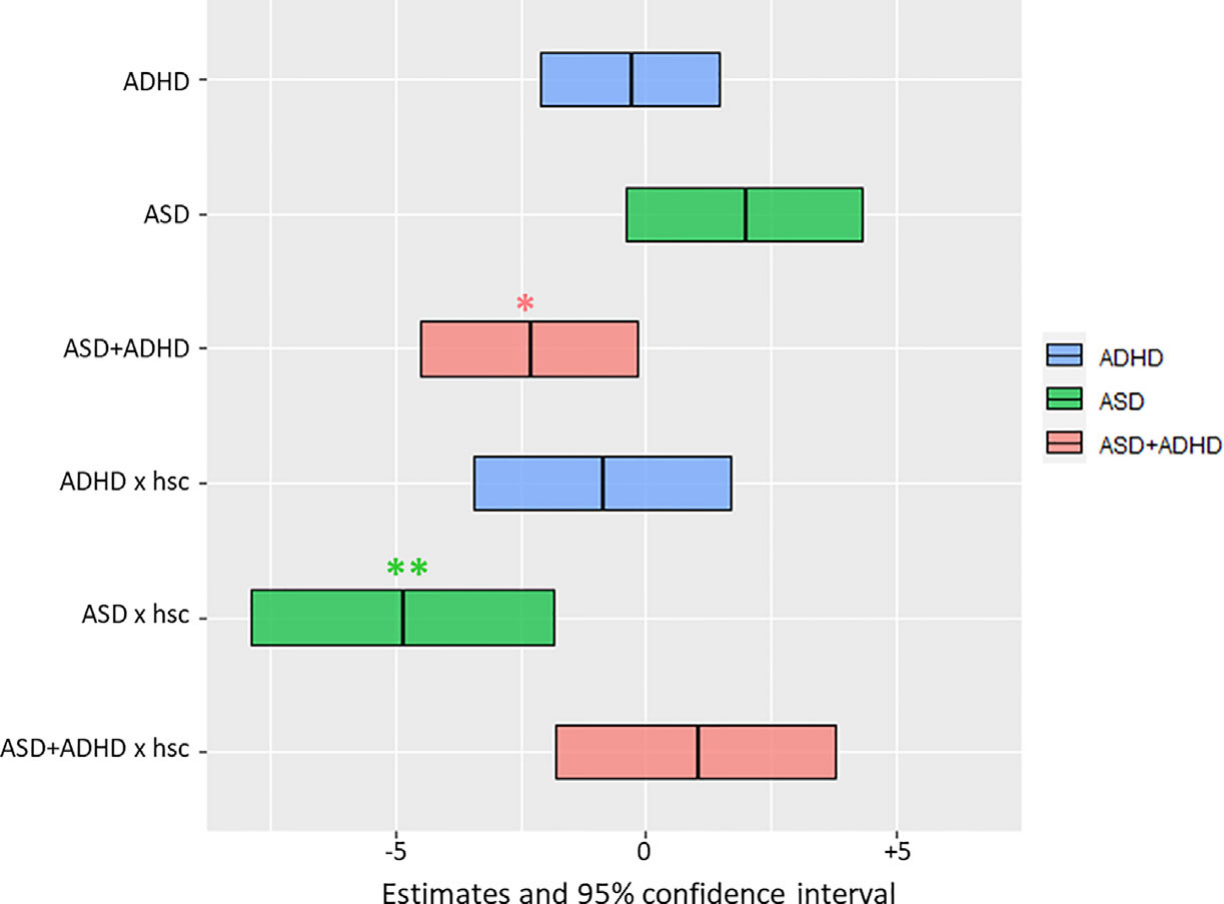
Results of the mixed effects analysis for the region of faces. Mean estimates of the fixed effects with 95% confidence interval are shown as center and border lines of each box, respectively. Their position on the x-axis (in seconds) relative to 0 is a measure of the statistical effect on the fixation duration to faces. Two fixed effects significantly reduce the fixation duration to faces, namely, the ASD + ADHD and the interaction term of ASD x high social complexity. Color coding is explained in the legend. *p < 0.05, **p < 0.01. Abbreviations: TD, Typically Developing; ADHD, Attention-Deficit/Hyperactivity Disorder; ASD, Autism Spectrum Disorder; ASD + ADHD, Autism Spectrum Disorder with comorbid Attention-Deficit/Hyperactivity Disorder; hsc, high social complexity; x, interaction between two effects.

In the mixed effects analysis, only the comorbid group showed significantly lower fixation durations on faces irrespective of social-complexity, with 2.3 s less (F-value = 4.65, p-value of *post-hoc* test = 0.032), while the two other groups did not differ compared to TD (see **Table 2**). For the ASD group there was a significant interaction with social-complexity, with 4.9 s shorter fixation durations to faces in stimuli with high social-complexity (F-value = 4.75, p-value of *post-hoc* test = 0.001, see **Table 2**). Previous training of social competence did not affect fixation duration on the face AOIs. Fixation estimates are visualized in **Figure 2**.

**Table 2:** Fixed effects parameter estimates for fixation duration on faces from 73 subjects.

Fixed effect	F value	Effect	Estimate	95% CI		SE	p-value
				Lower	Upper		
		(Intercept)	14.4	13.5	15.3	0.5	<.001
**Group**	4.65						
		- ADHD vs. TD	−0.3	−2.1	1.4	0.9	0.717
		- ASD vs. TD	2	−0.4	4.3	1.2	0.102
		- ASD + ADHD vs. TD	−2.3	−4.5	−0.2	1.1	0.032
**Group × social complexity**	4.75						
		ADHD vs. TD × high vs. low	−0.9	−3.4	1.6	1.3	0.493
		ASD vs. TD × high vs. low	−4.9	−7.8	−1.9	1.5	0.001
		ASD + ADHD vs. TD × high vs. low	1.0	−1.8	3.7	1.4	0.480
**Social complexity**	662.98	high vs. low	−13.7	−14.7	−12.6	0.5	<.001
**Total fixation duration**	28.64	(covariate)	0.1	0.1	0.2	0.03	<.001
**Previous social training**	1.59	yes vs. no	1.3	−0.7	3.4	1.1	0.212

TD, Typically Developing; ADHD, Attention-Deficit/Hyperactivity Disorder; ASD, Autism Spectrum Disorder; ASD + ADHD, comorbid group with ASD and ADHD; CI, Confidence Interval; SE, Standard Error; vs., versus; x, interaction between two effects.

For the first 5 and 10 s, analogous mixed effects analysis of fixations on faces was implemented. For the first 5 s, no group difference was evident. At 10 s, the total fixation duration on face AOIs across all groups was on average 2.6 ± 1.3 s for low versus 0.9 ± 0.7 s for high social-complexity stimuli. In the ASD + ADHD, the fixation duration on faces was significantly lower for both social-complexities by 0.3 s compared to the TD group (F-value = 4.1, p-value of *post-hoc* test = 0.015). Consistent with the results for the total run duration, the other clinical groups did not show any significant differences. The interaction observed between social-complexity and the ASD group for the total run duration, was at trend level at early gazing (F-value = 2.1, p-value of *post-hoc* test = 0.051). Tabular results of fixed effects parameter estimates for 5 and 10 s can be found in **Supplementary Tables 3** and **4**.

### Fixation Duration on Bodies and on Non-Social Elements

No significant group effects and no interactive effects were observed for the AOIs of bodies. Similar to the face AOI the fixation duration on high social complexity stimuli was distributed among the bodies of the four depicted persons (F-value = 173.9, p-value of *post-hoc* test < 0.001). The mixed effects analysis with the non-social elements AOI category did not show any group differences or any interactions of group with social complexity. There was a significant effect of social complexity, with participants fixating more on non-social elements in low social complexity stimuli (F-value = 118.3, p-value of *post-hoc* test < 0.001). Detailed fixed effects parameter estimates can be found in **Supplementary Tables 5** and **6**.

### Latency to First Face Fixation and Completion of Face Fixations

All four groups showed similar latencies to first face fixation (Group F_(3,283)_ = 1.0, p = 0.392; all contrasts versus TD: p-values non-significant) and no interactive effects with social complexity. By contrast, the latency to completion of face fixations was 5.7 s higher in the ASD + ADHD compared to the TD group (Group F_(3,141)_ = 3.49, p = 0.017, p-value of contrast 0.003). The other two clinical groups did not show prolonged latencies compared to the TD group.

## Discussion

The present study set out to investigate social visual attention in the neurodevelopmental disorders ASD, ADHD, and their comorbidity ASD + ADHD applying objective gaze measurements which are proposed to contribute to the development of valid biomarkers. To our knowledge, this is the first eye tracking study comparing social visual attention directly in all three clinical groups and TD controls.

In summary, the comorbid group showed significantly reduced social visual attention to faces in comparison to the control group. This effect was consistent across different levels of social complexity and after correction for their reduced total fixation duration and was detected using two representative eye tracking variables. Firstly, the fixation duration on faces was significantly reduced. Secondly, the time needed for the completion of face fixations was significantly prolonged, possibly reflecting a latency in the perception of the social configuration of interacting faces. Moreover, the significant effect of reduced fixation duration on faces was also evident at the early gaze behavior. Interestingly, there was an effect of social complexity on facial fixation duration in the ASD group but not in TD controls; ASD individuals looked less at faces in the socially complex images compared to the socially simple ones. Finally, participants with ADHD did not differ from controls in any of the gaze behavior parameters investigated. Taken together, reduced social visual attention was present in the comorbid group ASD + ADHD, while in the ASD group, it was reduced compared to TD controls when looking at scenes with high as opposed to low social complexity.

The present findings for the comorbid group are new, given that ASD cases have not been previously differentiated from the comorbidity cases in eye-tracking. Our findings are in line with recent non-eye-tracking studies of social cognition that consider this differentiation. Namely, in a study of visual and auditory emotion recognition, the comorbid group found to be somewhat more impaired than the “pure” groups in the speed of emotion recognition in both modalities (74). Elsewhere, autistic symptomatology, based on SRS and ADI-R, was found to be more pronounced in the ASD + ADHD in comparison to ASD and particularly regarding the social interaction subscale (ADI-R) (75). Such effects have been proposed to describe a more severe phenotype in ASD + ADHD (76). Other scenarios refer to comorbidity as a separate nosology (77) or propose the existence of subtypes of these two neurodevelopmental disorders (78). However, given their evolution of symptomatology across developmental ages (79), their genetic origins (80) and the recency of this discussion, more research directly investigating the comorbid ASD + ADHD group is needed to clarify this topic and replicate and extend our findings.

As highlighted in recent reviews on eye-tracking in ASD, social complexity is crucial for the performance of ASD subjects in social visual attention (37). However, only a handful of studies clearly report coexisting ADHD diagnosis. Therefore, findings from such samples should be interpreted with certain caution. Here, subjects with ASD-only were studied at different levels of social complexity and indeed there was a significant interaction between the ASD group and social complexity. This signifies that ASD participants spent less time on faces of people interacting with each other rather than on isolated faces. Yet, the time spent on faces across levels of social complexity was similar to the TD group, which replicates previous findings, where autistic children had the same fixation behavior, in terms of fixation time at faces, as TD children (33). Consequently, the conclusions concerning the importance of social complexity are partially replicated here. Moreover, adding to the existing conclusions, this study suggests that apart from social complexity, the existing comorbidity of ADHD could also play a crucial role in the performance of ASD in social visual attention. Thus, the timely need for reporting ADHD comorbidity as common practice in ASD research is underlined (81).

In the current analysis, latency to isolated faces in ASD participants was similar to TD, replicating previous findings. Particularly, in a comparable study including social images of different social complexity, children with ASD spent a similar amount of time as TD to first fixate to the face AOI (70). In our study, the latency of attention to a group of faces was additionally investigated and also introduced as a new measurement. Interestingly, the ASD + ADHD group required significantly longer until all four interacting faces were fixated for the first time when contemplating the socially complex scenes, suggesting they may have required longer in order to conceive the configuration of interacting people. Thus, the latency of attention to a group of faces, as presented here, could be meaningful for studying the apprehension of complex social interactions. Thus, it remains to be validated in future studies as a possible sensitive indicator of social visual attention deficits.

Apart from the clinical and theoretical implications of this study discussed above, the data analysis in this eye-tracking study offers various methodological advantages. Firstly, the type of linear regression model used allows for a modularly expandable introduction of explanatory variables, such as the status of previous social competence training and the covariate of total fixation duration. Secondly, with the mixed effects model, the impact of the explanatory variables on the fixation data is differentiated from the inter-individual gazing traits. This approach is essential in psychiatric research even more in the era of the dimensionality of disorders. (82). Thirdly, repeated measures, from two stimuli each, were nested in the two levels of social complexity (83). Finally, eye tracking offers measurable variables that are promising in social attention and are related to real-life social behaviors of neurodevelopmental disorders (84). In conjunction with robust statistical methods, here we compare objective eye tracking measurements for ASD, ADHD, and ASD + ADHD that could eventually provide potential candidates of valid biomarkers for differentiating these disorders. Recently, patterns of visual social attention which were obtained through gaze behavior were associated with caregiver-reported measures of social communication used in clinical trials (19). Thus, gaze measurements, as presented in our study, could serve as biomarkers for early diagnosis, monitoring and further care interventions as recommended (47).

Limitations of this study include moderate participant and stimulus sample sizes, which were partially addressed with the application of mixed effects models. Especially recruitment of ASD-only participants proved challenging. Applying thorough inclusion criteria on datasets, on the one hand ensured good data quality, on the other side caused additional exclusion of participants. Therefore, our study could be considered as a pilot study. In order to reduce the number of comparisons for the eye-tracking variables, the clinical groups were only compared to the control group. Nevertheless, these limitations call for independent replications with different participant and stimulus samples. The original stimuli will be made available upon request. Additionally, there was a male predominance in the clinical samples, which is yet in accordance with the known prevalence of ASD and ADHD.

In conclusion, to the best of our knowledge, this study investigates for the first time social visual attention using the eye tracking method to compare groups of ADHD, ASD, ASD + ADHD and TD participants. Our findings underscore the timely need to investigate social attention in the comorbid group in alignment with the current DSM-V guidelines. In addition, we emphasize that the existence of ADHD comorbidity should be considered as a crucial factor having impact on social visual attention in ASD and therefore its reporting should be a common practice in ASD research. Eye tracking measurements and Free Viewing of real-life social scenes as here presented, could contribute to the investigation of social attention, differentiation of ASD, ADHD, and ASD + ADHD and lead to the development of valid biomarkers. In this way, research on these neurodevelopmental disorders could enable an early diagnosis, gradually allow a thorough understanding of their social challenges, and promote an efficacious assistance to the population, with accurate monitoring and care interventions that will improve their quality of life.

## Data Availability Statement

All results presented in this study are included in the article/**Supplementary Material**.

## Ethics Statement

The studies involving human participants were reviewed and approved by Ethik-Kommission der Albert- Ludwigs-Universität Freiburg Freiburg, Germany. Written informed consent to participate in this study was provided by the participants' legal guardian/next of kin. Written informed consent was obtained from the individuals for the publication of any potentially identifiable images or data included in this article.

## Author Contributions

CI: data collection, data analysis, statistical analyses, and manuscript writing. DS: study design and data collection. MS: statistical analyses and manuscript writing. AR: manuscript writing. LT: manuscript writing. NS: manuscript writing. CF: manuscript writing. MB-S: study design and manuscript writing. GB: statistical analyses and manuscript writing. CK: study design and manuscript writing. All authors contributed to the article and approved the submitted version.

## Funding

This research was supported by a grant of the Research Commission of the Medical Faculty of the University of Freiburg (KLE1076/16) and the State Funded Doctoral Scholarship of Baden-Wuerttemberg. The article processing charge was funded by the University of Freiburg in the funding programme Open Access Publishing.

## Conflict of Interest

The authors declare that the research was conducted in the absence of any commercial or financial relationships that could be construed as a potential conflict of interest.
