# Supplementary material for: Comorbidity Matters: Social Visual Attention in a Comparative Study of Autism Spectrum Disorder, Attention-Deficit/Hyperactivity Disorder and Their Comorbidity
Source: Front Psychiatry. 2020 Sep 30;11:545567. doi: 10.3389/fpsyt.2020.545567 (PMC7555692; doi:10.3389/fpsyt.2020.545567)
Supplement: Supplementary file 1 [file Table_1.docx]

Supplementary Material

# Supplementary Data S1

# The content and scenery of the four stimuli was developed based on existing literature and the International Affective Picture System (IAPS) with participation of professional actors. Evaluation and selection from a large sample followed by experienced clinicians of the research group. The stimuli had to meet following prerequisites:

# – for the stimuli with high social complexity, the depicted persons should have eye contact.

# – there should be no more than one person without eye contact to the other persons.

# – the face expression should differ between the depicted persons.

# – there should be no extreme face expressions.

# – The interaction between the depicted persons should arouse interest for continuation of exploration. The depicted scene should thus contain a certain degree of ambiguity and should not be too easy to interpret.

# – The non-social elements should cover a similar area of the stimulus.

# Supplementary Tables

Supplementary Table 1. Reported comorbidities of included participants

|  | **ADHD** | **ASD** | **ASD+ADHD** |
| --- | --- | --- | --- |
| diurnal/nocturnal enuresis | 2 | 2 | 1 |
| adjustment disorder | 3 | - | 1 |
| childhood emotional disorder | 1 | - | - |
| moderate depressive episode without somatic symptoms | 1 | - | - |
| specific spelling disorder | 1 | - | - |
| somnambulism and chronic headaches | 1 | - | - |
| specific insect phobia | - | 1 | - |
| developmental dyspraxia | - | 1 | - |
| mixed receptive-expressive language disorder | - | 1 | - |
| chronic tic disorder | - | 1 | - |
| vocal tic disorder | - |  | 1 |
| obsessive compulsive disorder | - | 1 | - |
| expressive language disorder | - |  | 1 |

***Supplementary Table 1. Reported comorbidities of included participants.*** *ADHD: attention deficit/hyperactivity disorder, ASD: autism spectrum disorder, ASD+ADHD: autism spectrum disorder with comorbid attention deficit/hyperactivity disorder*

Supplementary Table 2. Fixed Effects parameter estimates for total fixation duration

|  |  | **95% Confidence Interval** | |  |  |
| --- | --- | --- | --- | --- | --- |
| **Effect** | **Estimate** | **Lower** | **Upper** | **SE** | **p-value** |
| (Intercept) | 102.8 | 100.3 | 105.4 | 1.3 | < .001 |
| ADHD vs. TD | -3.3 | -8.0 | 1.4 | 2.4 | 0.176 |
| ASD vs. TD | -4.9 | -11.2 | 1.5 | 3.2 | 0.138 |
| ASD+ADHD vs. TD | -6.2 | -11.9 | -0.5 | 2.9 | 0.038 |
| high vs. low social complexity | -3.6 | -5.4 | -1.8 | 0.9 | < .001 |
| Previous social training | -0.7 | -6.9 | 5.4 | 3.1 | 0.812 |
| ADHD vs. TD ✻ high vs. low social complexity | 3.7 | -0.6 | 8.1 | 2.2 | 0.096 |
| ASD vs. TD ✻ high vs. low social complexity | 2.5 | -2.7 | 7.7 | 2.7 | 0.354 |
| ASD+ADHD vs. TD ✻ high vs. low social complexity | 0.7 | -4.1 | 5.6 | 2.5 | 0.772 |

*Supplementary Table 2. Fixed Effects parameter estimates for total fixation duration from 73 subjects. TD: Typically Developing, ADHD: Attention Deficit/Hyperactivity Disorder, ASD: Autism Spectrum Disorder, ASD+ADHD: comorbid group with ASD and ADHD, SE: Standard Error, vs.: versus, ✻ : denotes interaction*

Supplementary Table 3. Fixed Effects parameter estimates for fixation duration on faces for the first 5 seconds

|  |  | **95% Confidence Interval** | |  |  |
| --- | --- | --- | --- | --- | --- |
| **Effect** | **Estimate** | **Lower** | **Upper** | **SE** | **p-value** |
| (Intercept) | 1.1 | 1 | 1.2 | 0.03 | < .001 |
| ADHD vs. TD | -0.03 | -0.2 | 0.1 | 0.1 | 0.587 |
| ASD vs. TD | 0.1 | -0.02 | 0.3 | 0.1 | 0.096 |
| ASD+ADHD vs. TD | 0.01 | -0.1 | 0.2 | 0.1 | 0.934 |
| high vs. low social complexity | -1.2 | -1.3 | -1.1 | 0.1 | < .001 |
| Total fixation duration | 0.2 | 0.1 | 0.2 | 0.03 | < .001 |
| Previous social training | 0.1 | -0.04 | 0.2 | 0.1 | 0.181 |
| ADHD vs. TD ✻ high vs. low social complexity | -0.01 | -0.3 | 0.2 | 0.1 | 0.933 |
| ASD vs. TD ✻ high vs. low social complexity | -0.4 | -0.6 | -0.1 | 0.2 | 0.02 |
| ASD+ADHD vs. TD ✻ high vs. low social complexity | -0.2 | -0.4 | 0.1 | 0.1 | 0.233 |

***Supplementary Table 3. Fixed Effects parameter estimates for fixation duration on faces for the first 5 seconds****. Abbreviations as in Supplementary Table 2.*

Supplementary Table 4. Fixed Effects parameter estimates for fixation duration on faces for the first 10 seconds

|  |  | **95% Confidence Interval** | |  |  |
| --- | --- | --- | --- | --- | --- |
| **Effect** | **Estimate** | **Lower** | **Upper** | **SE** | **p-value** |
| (Intercept) | 1.8 | 1.7 | 1.9 | 0.1 | < .001 |
| ADHD vs. TD | -0.1 | -0.3 | 0.1 | 0.1 | 0.335 |
| ASD vs. TD | 0.1 | -0.1 | 0.4 | 0.1 | 0.298 |
| ASD+ADHD vs. TD | -0.3 | -0.5 | -0.1 | 0.1 | 0.015 |
| high vs. low social complexity | -1.8 | -1.9 | -1.6 | 0.1 | < .001 |
| Total fixation duration | 0.1 | 0.1 | 0.2 | 0.03 | < .001 |
| Previous social training | 0.2 | 0.01 | 0.4 | 0.1 | 0.046 |
| ADHD vs. TD ✻ high vs. low social complexity | 0.05 | -0.3 | 0.4 | 0.2 | 0.802 |
| ASD vs. TD ✻ high vs. low social complexity | -0.4 | -0.9 | 0 | 0.2 | 0.051 |
| ASD+ADHD vs. TD ✻ high vs. low social complexity | 0.1 | -0.3 | 0.5 | 0.2 | 0.513 |

***Supplementary Table 4. Fixed Effects parameter estimates for fixation duration on faces for the first 10 seconds****. Abbreviations as in Supplementary Table 2.*

Supplementary Table 5. Fixed Effects Parameter Estimates for fixation duration on bodies

|  |  | **95% Confidence Interval** | |  |  |
| --- | --- | --- | --- | --- | --- |
| **Effect** | **Estimate** | **Lower** | **Upper** | **SE** | **p-value** |
| (Intercept) | 8.3 | 7.5 | 9.0 | 0.4 | < .001 |
| ADHD vs. TD | 0.2 | -1.3 | 1.7 | 0.8 | 0.755 |
| ASD vs. TD | -0.3 | -2.2 | 1.7 | 1.0 | 0.799 |
| ASD+ADHD vs. TD | 1.0 | -0.8 | 2.8 | 0.9 | 0.292 |
| high vs. low social complexity | -6.9 | -7.8 | -5.8 | 0.5 | < .001 |
| Total fixation duration | 0.04 | -0.005 | 0.1 | 0.03 | 0.082 |
| Previous social training | -0.6 | -2.3 | 1.1 | 0.9 | 0.497 |
| ADHD vs. TD ✻ high vs. low social complexity | -0.2 | -2.7 | 2.2 | 1.2 | 0.863 |
| ASD vs. TD ✻ high vs. low social complexity | -0.3 | -3.2 | 2.6 | 1.5 | 0.855 |
| ASD+ADHD vs. TD ✻ high vs. low social complexity | -0.3 | -2.9 | 2.4 | 1.4 | 0.855 |

Supplementary Table 5. Fixed Effects Parameter Estimates for fixation duration on bodies. Abbreviations as in Supplementary Table 2.

Supplementary Table 6. Fixed Effects Parameter Estimates for fixation duration on non-social elements

|  |  | **95% Confidence Interval** | |  |  |
| --- | --- | --- | --- | --- | --- |
| **Effect** | **Estimate** | **Lower** | **Upper** | **SE** | **p-value** |
| (Intercept) | 6.7 | 6.1 | 7.2 | 0.3 | < .001 |
| ADHD vs. TD | -0.2 | -1.2 | 0.8 | 0.5 | 0.647 |
| ASD vs. TD | -0.2 | -1.5 | 1.2 | 0.7 | 0.815 |
| ASD+ADHD vs. TD | 0.1 | -1.1 | 1.3 | 0.6 | 0.886 |
| high vs. low social complexity | -4.5 | -5.4 | -3.7 | 0.4 | < .001 |
| Total fixation duration | 0.08 | 0.04 | 0.1 | 0.02 | <.001 |
| Previous social training | -0.4 | -1.7 | 0.9 | 0.6 | 0.543 |
| ADHD vs. TD ✻ high vs. low social complexity | 0.5 | -1.5 | 2.4 | 1.0 | 0.642 |
| ASD vs. TD ✻ high vs. low social complexity | 1.3 | -1.0 | 3.6 | 1.2 | 0.270 |
| ASD+ADHD vs. TD ✻ high vs. low social complexity | 0.7 | -1.4 | 2.9 | 1.1 | 0.506 |

*Supplementary Table 6. Fixed Effects Parameter Estimates for fixation duration* *on non-social elements. Abbreviations as in Supplementary Table 2.*
